# Novel and Novel De Novo Mutations in *NTRK1* Associated With Congenital Insensitivity to Pain With Anhidrosis: A Case Report

**DOI:** 10.1097/MD.0000000000000871

**Published:** 2015-05-21

**Authors:** Qingli Wang, Shanna Guo, Guangyou Duan, Guifang Xiang, Ying Ying, Yuhao Zhang, Xianwei Zhang

**Affiliations:** From the Department of Anesthesiology, Tongji Hospital, Tongji Medical College, Huazhong University of Science and Technology, Wuhan, Hubei, China (QW, SG, GD, GX, YY, YZ, XZ); and Department of Anesthesiology, Wuhan General Hospital of Guangzhou Military, Wuhan, China (QW).

## Abstract

Congenital insensitivity to pain with anhidrosis (CIPA) is a very rare autosomal recessively inherited disorder. The main clinical features of the disorder consist of absence of reactions to noxious stimuli and inability to sweat under any conditions.

In this case report, a 3-year-old Chinese boy diagnosed with CIPA presented with the core features of CIPA, including insensitivity to noxious stimuli, self-mutilation, inability to sweat, and developmental delay. Clinical and genetic analyses were conducted on the affected boy.

Sequencing analysis revealed an inherited novel mutation, c.1635G>C, and a novel de novo mutation, c.2197G>A, in the *NTRK1* gene. In silico studies suggested that the mutations described are detrimental to the function of the protein encoded by the *NTRK1* gene.

The two novel mutations described here widen the genetic spectrum of CIPA, and this knowledge will benefit studies addressing this disease and pain medicine in the future.

## INTRODUCTION

Congenital insensitivity to pain with anhidrosis (CIPA; MIM #256800), also referred to as hereditary sensory and autonomic neuropathy type 4 (HSAN4), is a rare autosomal recessively inherited disorder, which was first described by Swanson in 1963.^1^ The core clinical features of the disorder consist of absence of reactions to noxious stimuli and inability to sweat under any conditions, with or without variable mental retardation. Secondary consequences include self-mutilating behavior, multiple injuries, repeated painless fractures, Charcot arthropathy, osteomyelitis, and repeated fever. Furthermore, some patients also present with delayed developmental milestones.^2^

Neurotrophic tyrosine kinase receptor type 1 (*NTRK1*) is considered the most likely candidate gene for CIPA, although involvement of other genes is a possibility.^3–5^*NTRK1* encodes a high-affinity receptor for nerve growth factor (NGF). Upon binding to NGF, NTRK1 undergoes autophosphorylation and activates the mitogen-activated protein kinase pathway, thereby regulating growth, survival, and differentiation of sympathetic and selected sensory neurons.^2^ Accordingly, histological studies of CIPA patients revealed defects in neurons, the growth of which is regulated by NGF, leading to complete absence of small-diameter non-myelinated fibers, mild loss of small-diameter myelinated fibers, and loss of innervation of eccrine sweat glands.^6–8^ Recent studies have also revealed that neurons responsive to NGF are involved in brain–immune–endocrine interactions relevant to pain, itch, and inflammation.^9,10^ Thus, targeting the molecular mechanisms of NGF-NTRK1 signal transduction may aid in the development of novel analgesic methods.

To date, approximately 50 loss-of-function *NTRK1* variants have been associated with CIPA (http://www.molgen.ua.ac.be/CMTMutations/); however, CIPA is a rare disease, and further genetic and clinical data are needed to facilitate its diagnosis. In China, only a few CIPA cases have been reported.^11^ In this paper, we described a Chinese CIPA patient with novel and novel de novo *NTRK1* mutations. To our knowledge, this is the first study describing a de novo *NTRK1* mutation in CIPA patients.

## PATIENT AND METHODS

### Patient

This study was approved by the institutional ethics committee of Tongji Hospital, Tongji Medical College, Huazhong University of Science and Technology (Approved ID: 20130501). Written informed consent was obtained from the patient's parents before the study.

The patient was a 3-year-old boy diagnosed with CIPA. We investigated the patient, his parents, and his brother. The clinical data were obtained from clinician's interviews and chart reviews. Measurement of thresholds to noxious thermal and mechanical stimuli, intradermal histamine test, and perspiration test were performed to confirm the CIPA diagnosis. Identical tests were conducted on the patient's parents to generate control data. Nerve biopsies were not considered necessary because the procedure would be harmful to the patient and would be of minimal benefit to the study.

### Genetic Analysis

Peripheral blood was obtained from the patient, his parents, and his brother. Genomic DNA was extracted from the blood samples by using the TIANamp Genomic DNA Kit (Tiangen Biotech, Beijing, China), according to the manufacturer's protocol.

Seventeen exons and intron–exon boundaries of the transcript variant 2 of *NTRK1*, currently believed to be the only isoform expressed in neuronal tissues,^12–14^ were amplified by polymerase chain reaction in the presence of primers used in previous reports.^15,16^ Direct bidirectional sequencing of the amplified DNA fragments was performed using an ABI3730XL automated sequencer (Applied Biosystems, Foster City, CA). The resulting sequences were analyzed using Chromas software (version 2.22) and compared with the current reference sequence for *NTRK1* in the NCBI database (NM_002529.3), corresponding to the amino sequence NP_002520.2. Sequencing reactions and alignments to the reference human transcriptome were repeated twice. Subsequently, sequence variants were retrieved from dbSNP135 and were tested in 200 chromosomal sequences from a control cohort of 100 healthy Han Chinese individuals.

### In Silico Studies

Initially, the amino acid (AA) substitution score was assessed in the context of the physicochemical environment with software available online (http://www.russell.embli-heidelberg.de/aas/) by obtaining a detailed topological annotation for NTRK1 from UniProt (http://www.uniprot.org). Subsequently, multiple sequence alignments were conducted to evaluate the conservation of the AA sequence. Finally, the impacts of AA substitution were predicted by the computational algorithm Polymorphism Phenotyping v2 (PolyPhen-2; http://genetics.bwh.harvard.edu/pph2/).

## RESULTS

### Clinical Data

**FIGURE 1:**
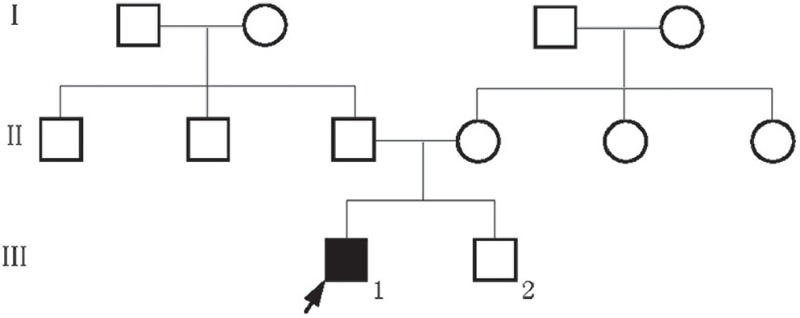
Pedigree of the described family.

**FIGURE 2:**
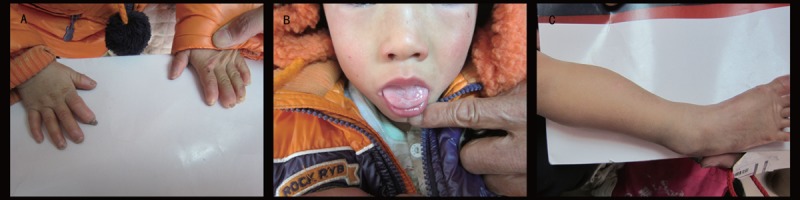
(A) Bitten fingernails, (B) defective tongue tip, and (C) malformed right shinbone observed in the 3-year-old Chinese boy diagnosed with congenital insensitivity to pain with anhidrosis.

The proband was a 3-year-old boy delivered spontaneously after a full-term pregnancy, there were no known exposures to harmful conditions during pregnancy, and his parents were non-consanguineous. Other members in the proband's family, his parents and a younger brother, were unaffected (Figure 1). The patient had typical CIPA features such as insensitivity to noxious stimuli, inability to sweat, and various complications. An initial sign of the proband was the absence of reaction to injections in early infancy, and his insensitivity to pain became gradually apparent after absence of reactions to harmful events were observed (Figure 2). He bit his fingers and tongue as his teeth were erupting. His right tibia was fractured at age 1 without any pain, an event that lead to a malformed shinbone. Three days after birth, the proband suffered his first fever. Although various clinical tests recorded normal results, no drugs were effective against the fever; hence, the body temperature was decreased by physical cooling. His parents noticed his anhidrosis after he experienced a few episodes of fever. He was diagnosed with CIPA at age 1, based on clinical features. Development was also abnormal, since the proband could neither walk independently nor say a complete sentence at the age 3. His hair was thin.

### Genetic Analysis

**FIGURE 3:**
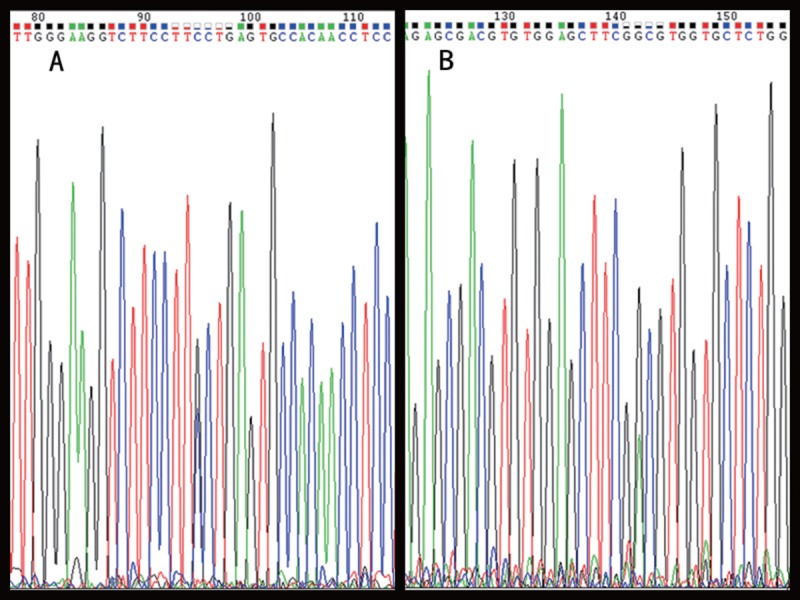
Sequencing traces of c.1635G>C and c.2197G>A in *NTRK1* of the proband.

Peripheral blood samples were obtained from the proband, his parents, and his brother. All 17 exons and intron–exon boundaries of *NTRK1* were amplified successfully. Sequencing analysis of *NTRK1* in the proband revealed 2 novel compound heterozygous missense mutations (Figure 3). Novel missense mutations were detected in exon 13 (c.1635G>C; p.Ala527Pro) and in exon 16 (c.2197G>A; p.Gly714Asp). Both the novel missense mutations in the proband were heterozygous. Genetic analysis of family members revealed that the p.Ala527Pro mutation was inherited from the father, who was heterozygous for the mutation, whereas the mother was wild type. The p.Gly714Asp substitution was a de novo mutation because neither parent carried the mutation. The brother did not carry any of the mutations. The 2 substitutions were not observed in the 100 healthy Han Chinese control subjects.

**FIGURE 4:**
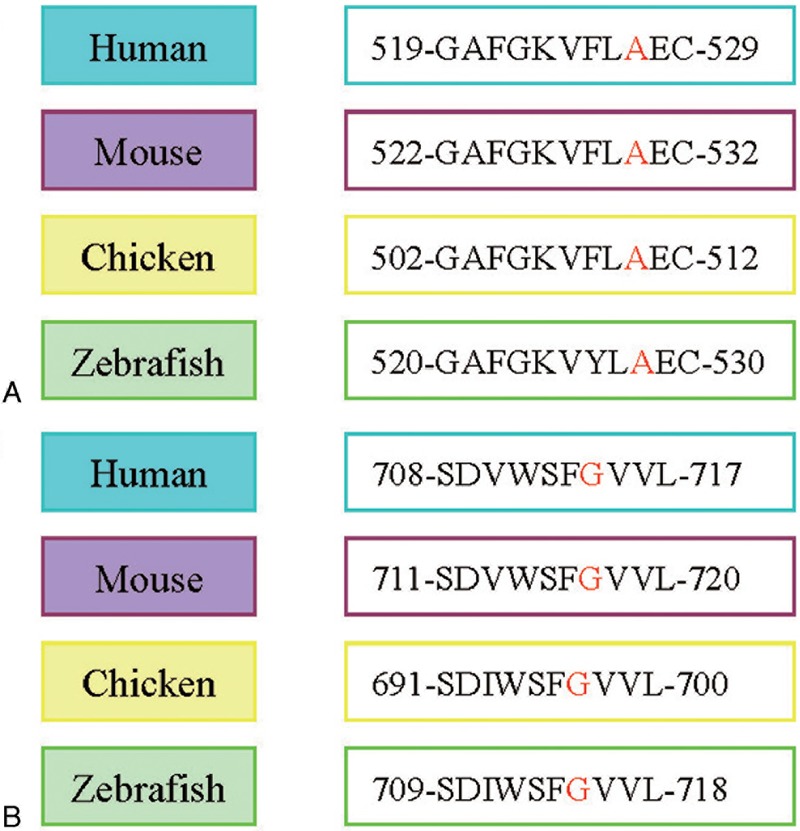
Ala527 and Gly714 in *NTRK1* are evolutionarily conserved from zebrafish to humans.

Both the AA substitution scores of p.Ala527Pro and p.Gly714Asp were “0,” representing “neutral.” Multiple sequence alignment revealed that Ala527 and Gly714 in NTRK1 are evolutionarily conserved from zebrafish to humans (Figure 4). Analysis conducted with PolyPhen-2 predicted that both the p.Ala527Pro and p.Gly714Asp substitutions were detrimental to protein function.

## DISCUSSION

CIPA is an autosomal recessively inherited disorder, resulting from the presence of 2 *NTRK1* pathogenic variants. Usually, 1 pathogenic variant is inherited from each parent, and in a few cases, both pathogenic variants are inherited from a single parent. In this study, we described an inherited novel mutation, c.1635G>C (Ala527Pro), and a novel de novo mutation, c.2197G>A (Gly714Asp), in *NTRK1*.

Three transcript variants of human *NTRK1* have been reported. Transcript variant 2, which is divided into 17 exons and 16 introns, is expressed specifically in neuron tissues; and isoform *NTRK1-II* (identifier: P04629-1) was chosen as the ‘canonical’ NTRK1 sequence. The mRNA of transcript variant 2 (NM_002529.3) encodes a protein of 796 AA residues (NP_002520.2). NTRK1 is a transmembrane protein composed of an extracellular, a transmembrane, and an intracellular domain.^17^ The 3 domains are encoded by exons 1 to 9, exons 10 to 11, and exons 11 to 17, respectively. The intracellular domain comprises a juxtamembrane region, a tyrosine kinase domain (TKD), and a short carboxyl-terminal tail. The TKD encoded by exons 13 to 17 is required for signal transduction.^18^ Sequence annotation in the UniProt database shows that natural *NTRK1* variations associated with CIPA are mainly located in the TKD domain (http://www.uniprot.org/uniprot/P04629#P04629). Similarly, the 2 novel *NTRK1* mutations presented in this paper were also located in the TKD domain.

Although the substitution scores for the Ala527Pro and Gly714Asp mutations were assessed as neutral under selected intracellular environment conditions, the possibility of the mutation being detrimental to protein function was not excluded.^19^ Alanine is an aliphatic AA that usually mediates substrate recognition or specificity, particularly in interactions with non-reactive atoms. The side chain of the AA proline is a cyclical structure of pentagonal shape, connected to the protein backbone by 2 individual bonds. Because of the structural constraints introduced by proline in the polypeptide chain, this AA is rarely present in the active or interacting sites of proteins. Thus, p.Ala527Pro was unlikely to be a neutral substitution in this study. Similarly, the p.Gly714Asp substitution had the potential to be harmful, given that glycine lacks a side chain, allowing it to confer conformational flexibility on the polypeptide chain, a function that cannot be carried out by any other AA.

Further bioinformatics analyses predicted that these mutations might cause CIPA. Alignments with multiple sequences showed that Ala527 and Gly714 of NTRK1 are evolutionarily conserved from zebrafish to humans, suggesting that mutation at these sites are detrimental to NTRK1 function. Accordingly, PolyPhen-2 analysis revealed that p.Ala527Pro and p.Gly714Asp were predicted as probably damaging to protein function, strongly suggesting that both mutations cause defects in NTRK1, leading to CIPA in the study subject.

To our knowledge, this is the first report to identify a de novo mutation causing CIPA; our findings provide novel insight into the genetic basis of CIPA and reiterate the importance of a careful genetic counseling strategy. These kind of rare human genetic disorders provide opportunities to explore pathological conditions in humans and the underlying normal biological processes. The molecular pathophysiology of CIPA can provide important clues about the biological processes of pain in our species and insights into the mechanisms that underlie persistent or chronic pain syndromes.^2^

In conclusion, we presented novel inherited and de novo heterozygous missense mutations in *NTRK1* of a Chinese patient with CIPA. These mutations widen the genetic spectrum of CIPA, and provide data for further screening of founder *NTRK1* mutations in Chinese Han patients with CIPA. Knowledge of the molecular mechanisms of NGF-NTRK1 signal transduction promises to lead to the development of novel and effective analgesic approaches.
